# Inequalities in glycemic and multifactorial cardiovascular control of type 2 diabetes: The Heart Healthy Hoods study

**DOI:** 10.3389/fmed.2022.966368

**Published:** 2022-12-07

**Authors:** Sara Ares-Blanco, Elena Polentinos-Castro, Francisco Rodríguez-Cabrera, Pedro Gullón, Manuel Franco, Isabel del Cura-González

**Affiliations:** ^1^Federica Montseny Health Centre, Gerencia Asistencial Atención Primaria, Servicio Madrileño de Salud, Madrid, Spain; ^2^Medical Specialties and Public Health, School of Health Sciences, University Rey Juan Carlos, Alcorcón, Spain; ^3^Instituto de Investigación Sanitaria Gregorio Marañón, Madrid, Spain; ^4^Primary Care Research Unit, Gerencia de Atención Primaria, Servicio Madrileño de Salud, Madrid, Spain; ^5^Health Services Research on Chronic Patients Network (REDISSEC and RICAPPS), Instituto de Salud Carlos III, Madrid, Spain; ^6^Unidad Docente Medicina Preventiva y Salud Pública, National School of Public Health, Madrid, Spain; ^7^Public Health and Epidemiology Research Group, Universidad de Alcalá, Alcala de Henares, Spain; ^8^Department of Epidemiology, Johns Hopkins Bloomberg School of Public Health, Baltimore, MD, United States

**Keywords:** type 2 diabetes, sex, primary health care, social determinants of health, decision making

## Abstract

**Aim:**

This study aimed to analyze glycemic control and multifactorial cardiovascular control targets in people with type 2 diabetes (T2DM) in primary care according to sex and socioeconomic status (SES).

**Materials and methods:**

This is an observational, cross-sectional, and multicenter study. We analyzed all the patients with T2DMM aged between 40 and 75 years in Madrid city (113,265) through electronic health records from 01 August 2017 to 31 July 2018. SES was defined by an area-level socioeconomic index stratified by quintiles (1st quintile: more affluent).

**Outcomes:**

Outcomes included glycemic control (HbA1c ≤ 7%), 3-factor cardiovascular control [HbA1c ≤ 7%, blood pressure (BP), < 140/90 mmHg, LDL < 100 mg/ml] and 4-factor control [HbA1c ≤ 7%, blood pressure (BP) < 140/90 mmHg, LDL < 100 mg/ml, and BMI < 30 kg/m^2^]. Multilevel logistic regression models analyzed factors associated with suboptimal glycemic control.

**Results:**

In total 43.2% were women. Glycemic control was achieved by 63% of patients (women: 64.2% vs. men: 62.4%). Being more deprived was associated with suboptimal glycemic control (OR: 1.20, 95% CI: 1.10–1.32); however, sex was not related (OR: 0.97, 95% CI: 0.94–1.01). The optimal 3-factor control target was reached by 10.3% of patients (women: 9.3% vs. men: 11.2%), especially those in the 5th quintile of SES. The 4-factor control was achieved by 6.6% of the sample. In the 3-factor control target, being women was related to the suboptimal 3-factor control target (OR: 1.26, 95% CI: 1.19– 1.34) but only belonging to SES 4th quintile was related to the unachieved target (OR: 1.47, 95% CI: 1.04–2.07).

**Conclusion:**

Suboptimal glycemic control was associated with being less affluent and suboptimal 3-factor control target was associated with being women.

## Introduction

It is estimated that type 2 diabetes (T2DM) affects 463 million people worldwide (1). T2DM increases with age and also depends on social factors, such as education, income, neighborhood, or socioeconomic status (SES) (2). Patients with T2DM have an increased risk of mortality for all other conditions in comparison to patients without diabetes (3), especially in women (4).

To achieve optimal control of this condition, clinical guidelines recommend maintaining HbA1c of < 7%, blood pressure of ≤ 140/90 mmHg (BP), and a body mass index of < 30 kg/m^2^ (BMI). The weight goal differs among the guidelines, but it is recommended to avoid obesity and lose weight if overweight (5, 6). The LDL cholesterol target has changed over the years based on studies that addressed it in the cardiovascular risk factor context. Although the objectives are clear, only two-thirds of patients can reach the HbA1c goal (7, 8). Some studies found that patients whose HbA1c is outside the range of 6–8% have more cardiovascular complications (9), more visits to the family doctor (likely a proxy for more complex disease) (10), more admissions to the hospital resulting from complications (11), and higher mortality than those with HbA1c levels inside this range (12).

In the past, glycemic control was focused on achieving a single target defined by HbA1c <7%. Recently, some studies have described not only glycemic control but also the cardiovascular multifactorial control targets of HbA1c <7%, BP ≤140/90 mmHg, and LDL <100 mg/dl (multifactorial control targets) (13–16). Less information is known to determine which single target could help T2DM management. Wan et al. have suggested that LDL targets alone could decrease cardiovascular disease risk among patients with T2DM (16). In the past years, patient demographics such as sex and SES have been added to this approach because both determinants influence both healthy behaviors and access to the health system (17–19). Health inequalities have been observed in those living in more socioeconomically deprived areas as they were less likely to attain glycemic control and had more T2DM complications (18).

The Spanish National Health System (NHS) provides first-contact, comprehensive, continuous, and coordinated care, which is free at the point of care for a defined population served by primary care centers. Every citizen in Spain is assigned a family physician. The T2DM is managed by the family physicians who are responsible for delivering and coordinating patient care. Electronic health record has been used in the country for over 20 years in the public sector and the private sector. The Spanish NHS provides care through 17 counties; each county has its own electronic health record. All the counties share data with the NHS to elaborate on national data.

This study aimed to analyze glycemic control and multifactorial cardiovascular control targets regarding sex and socioeconomic status (SES) in type 2 diabetes in primary care.

## Materials and methods

### Study design

This is an observational, population-based, cross-sectional study. The Heart Healthy Hoods (HHH) project studies the association between the urban environment, cardiovascular health, and inequities in the whole of Madrid city (Spain) (20). HHH project gathered data through the electronic health records of 128 primary care practices in Madrid city (Spain).

### Participants

We analyzed all the patients with type 2 diabetes aged between 40 and 75 years and having at least one measure of HbA1c during 1 year from 01 August 2017 to 31 July 2018 registered in the primary care electronic health record of Madrid Public Health System. A flowchart describing the inclusion and exclusion of participants is shown in Supplementary Figure 1.

### Variables

Sociodemographic variables were recorded (sex and age). The area-level socioeconomic status index (MEDEA index) was assessed on a combination of four census indicators, namely, unemployment, low education level, the percentage of people who are manual workers, and those who are working in temporary jobs in relation to the employed population (21). Patients were grouped by quintiles of the socioeconomic index according to their neighborhood: the 1st quintile (less deprived) and the 5th quintile (more deprived).

According to the guidelines, we defined model 1 (glycemic control) as HbA1c ≤ 7% (53 mmol/mol). Model 2 (3-factor control) was defined by HbA1c ≤ 7%, BP < 140/90 mmHg, and LDL < 100 mg/ml, and model 3 (4-factor control) was defined as HbA1c ≤ 7%, BP < 140/90 mmHg, LDL < 100 mg/ml, and body mass index (BMI) < 30 kg/m^2^.

The clinical data included the duration of T2DM (years), cardiovascular risk factors (smoking, hypertension, dyslipidemia, and obesity defined as BMI ≥ 30 kg/m^2^), and cardiovascular complications (ischemic heart disease, stroke, peripheral vascular disease, chronic renal failure, diabetic nephropathy, and diabetic retinopathy). In addition, estimated glomerular filtration rate (calculated with CKD-EPI values and MDRD4) and albuminuria were collected. Chronic renal disease was defined as kidney damage or glomerular filtration rate (GFR) < 60 ml/min/1.73 m^2^ for 3 months or more (22). Albuminuria was defined as an albumin-to-creatinine ratio >30 mg/g in two of three spot urine specimens (22). Laboratory results were estimated as the arithmetic mean of the individual determinations during the 1 year for those who had more than one measurement.

Characteristics of the 128 primary care practices in Madrid city included the number of family doctors and nurses, the daily consultation rates of family doctors and nurses (patients/day), and the population size assigned to the primary health center for family doctors and nurses (Supplementary Table 1).

### Statistical analyses

All patient and practice characteristics were summarized using descriptive statistics (proportions and means, standard deviations or medians, and interquartile ranges, when appropriate based on the distribution).

Categorical variables (good glycemic or 3-factor and 4-factor control targets) were presented as percentages and compared using the χ^2^ test and the Student’s *t*-test or corresponding non-parametric tests for continuous variables.

Several variables potentially associated with factor controls were assessed using multilevel logistic regression analysis, taking into account the aggregation of data by cluster (patient: first level, primary care practice: second level), adjusted by age, sex, and SES index. Results were expressed as odds ratios (OR) and 95% CI. All the tests were conducted at a significance level of 0.05. The analysis was performed using STATA 15.1 and RStudio 16.0.

## Results

### Study population characteristics

Of the 3.22 million inhabitants in Madrid city, the Heart Healthy Hoods study analyzed individuals aged between 40 and 75 years (1.42 million). Among those, 113,265 had T2DM, of whom 68,535 (60.5%) had at least one check-up performed within the previous 12 months. Comparisons between those with and without an HbA1c measured in the last year are available in Supplementary Table 2.

The mean age was 62.7 ± 8.8 years, women comprised 43.2% of the total, and 41% of the population were classified in the lowest groups (4th quintile and 5th quintile of SES). The demographic and clinical characteristics of the study population are shown in Table 1 according to their achievement of the glycemic target. Patients who achieved glycemic control were slightly older and suffered more hypertension and obesity than those who did not achieve it; however, they had fewer T2DM complications than those with HbA1c >7%. The characteristics of cardiovascular factors stratified by sex can be found in Supplementary Table 3.

**TABLE 1:** General characteristics of the population according to their achievement of the glycemic target.

Patient characteristics	All	Patients with HbA1c ≤ 7%	Patients with HbA1c > 7%	*P*-value
N	68,535	43,296	25,239	
Age (years)*	62.7 (8.8)	63.2 (8.6)	61.8 (9.1)	<0.001
Duration of T2DM (years)*	9.40 (6.0)	8.6 (5.6)	10.9 (6.3)	<0.001
Foreigners **	4,111 (6.0)	2,219 (5.1)	1,892 (7.5)	<0.001
Men**	38,955 (56.8)	24,307 (56.1)	14,648 (58.04)	<0.001
Women**	29,580 (43.2)	18,989 (43.9)	10,591 (41.9)	
Socioeconomic status index**:				<0.001
1st quintile, least deprived	14,764 (21.6)	9,731 (22.6)	5,033 (20.0)	
2nd quintile	12,405 (18.2)	8,042 (18.7)	4,363 (17.3)	
3rd quintile	13,110 (19.2)	8,388 (19.5)	4,722 (18.8)	
4th quintile	15,393 (22.6)	9,427 (21.9)	5,966 (23.7)	
5th quintile, more deprived	12,568 (18.4)	7,503 (17.4)	5,065 (20.1)	
Cardiovascular Risk Factors**:				
Tobacco	4,799 (18.0)	2,910 (17.4)	1,889 (18.9)	0.002
Obesity	18,407 (46.0)	11,052 (60.0)	7,355 (39.9)	<0.001
Dyslipidemia	43,135 (62.9)	27,420 (63.3)	15,715 (62.3)	0.005
Hypertension	43,384 (63.3)	27,628 (63.8)	15,756 (36.3)	<0.001
T2DM Complications**:				
Coronary heart disease	7,057 (10.3)	4,227 (9.8)	2,830 (11.2)	<0.001
Stroke	3,908 (5.7)	2,426 (5.6)	1,482 (5.9)	0.14
Peripheral arteriopathy	3,212 (4.7)	1,818 (4.2)	1,394 (5.5)	<0.001
Chronic renal disease	2,123 (3.1)	1,311 (3.0)	812 (3.2)	0.17
Diabetic nephropathy	2,232 (14.4)	1,159 (11.7)	1,073 (19.4)	<0.001
Retinopathy	2,127 (3.1)	898 (2.1)	1,229 (4.9)	<0.001

*Mean (standard deviation).

**n (%).

SBP, systolic blood pressure; DBP, diastolic blood pressure; BMI, body mass index; SES, socioeconomic status index.

### Glycemic control, 3-factor control, 4-factor control by sex and socioeconomic status index

**FIGURE 1:**
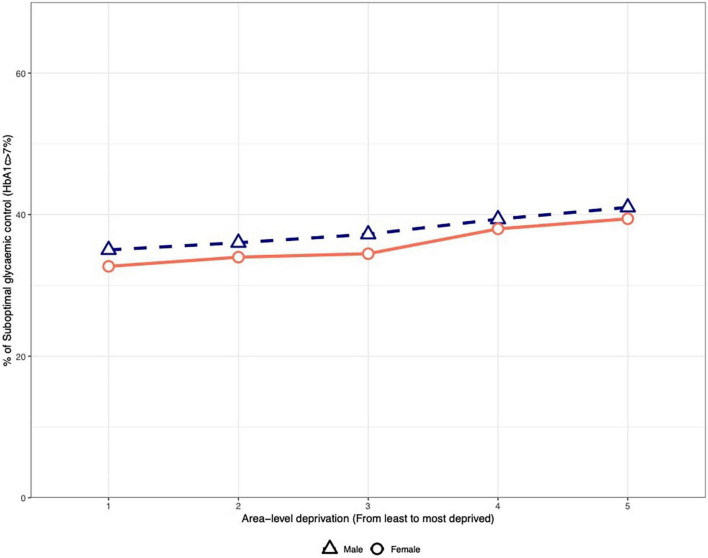
Suboptimal glycemic control based on the socioeconomic status index and sex.

Glycemic control was achieved in 63.2% of the population, with 64.2% of the female patients achieving it compared to 62.4% of male patients (*p* < 0.001) (Tables 2, 3). The proportion of patients who achieved glycemic control decreased from the 1st quintile to the 5th quintile of SES in both sexes. Men had lower rates of control in all the quintiles; however, the differences were small (Figure 1).

**TABLE 2:** Optimal control targets and complications in men based on a socioeconomic status index.

	All	1^st^ quintile n: 8,861	2^nd^ quintile n: 7,174	3^rd^ quintile n: 7,174	4^th^ quintile n: 8,625	5^th^ quintile n: 6,800	*P*-value
Optimal control*							
HbA1c	24,307 (62.4)	5,758 (65.0)	4,589 (64.0)	4,603 (62.8)	5,230 (60.6)	4,010 (59.0)	<0.001
HbA1c, BP, LDL	3,099 (11.1)	681 (11.1)	617 (11.4)	641 (12.3)	442 (7.9)	703 (13.4)	<0.001
HbA1c, BP, LDL, BMI	1,470 (7.5)	326 (6.6)	301 (6.5)	286 (6.3)	225 (4.2)	327 (7.3)	<0.001
Complications*							
Ischemic heart disease	5,474 (14.1)	1,348 (15.2)	964 (13.4)	1,030 (14.0)	1,210 (14.0)	904 (13.3)	0.004
Stroke	2,479 (6.4)	613 (6.9)	422 (5.9)	436 (5.9)	561 (6.5)	439 (6.5)	0.042
Peripheral arteriopathy	2,538 (6.5)	548 (6.2)	453 (6.3)	493 (6.7)	559 (6.5)	473 (7.0)	0.31
Diabetic nephropathy	1,564 (16.9)	335 (17.2)	264 (15.9)	291 (16.1)	378 (16.2)	292 (20.6)	0.002
Chronic renal disease	1,290 (3.3)	293 (3.3)	208 (2.9)	255 (3.5)	295 (3.4)	232 (3.4)	0.29
Retinopathy	1,298 (3.3)	280 (3.2)	237 (3.3)	241 (3.3)	242 (2.8)	292 (4.3)	<0.001

*Total and %.

**TABLE 3:** Optimal control targets and complications in women based on a socioeconomic status index.

	All	1^st^ quintile n:5,903	2^nd^ quintile n:5,231	3^rd^ quintile n:5,777	4^th^ quintile n:6,768	5^th^ quintile n:5,768	*P*-value
Optimal control*							
HbA1c	18,989 (64.2)	3,973 (67.3)	3,453 (66.0)	3,785 (65.5)	4,197 (62.0)	3,493 (60.6)	<0.001
HbA1c, BP, LDL	2,028 (9.2)	404 (9.5)	426 (10.4)	412 (9.7)	287 (6.3)	487 (10.6)	<0.001
HbA1c, BP, LDL, BMI	896 (5.5)	151 (5.2)	187 (6.6)	209 (6.6)	129 (3.4)	213 (6.1)	<0.001
Complications*							
Ischemic heart disease	1,583 (5.4)	293 (5.0)	273 (5.2)	295 (5.1)	383 (5.7)	333 (5.8)	0.21
Stroke	1,429 (4.8)	272 (4.6)	257 (4.9)	273 (4.7)	322 (4.8)	299 (5.2)	0.65
Peripheral arteriopathy	674 (2.3)	135 (2.3)	137 (2.6)	130 (2.3)	151 (2.2)	115 (2.0)	0.30
Diabetic nephropathy	668 (10.7)	114 (10.6)	116 (11.5)	150 (10.7)	159 (9.3)	127 (13.0)	0.054
Chronic renal disease	833 (2.8)	158 (2.7)	122 (2.3)	177 (3.1)	198 (2.9)	172 (3.0)	0.13
Retinopathy	829 (2.8)	138 (2.3)	136 (2.6)	153 (2.6)	190 (2.8)	206 (3.6)	<0.001

*Total and %.

When we addressed T2DM as a 3-factor control target (HbA1c ≤ 7%, LDL < 100 mg/dl, and BP < 140/90 mmHg), we found that 9.2% of women and 11.1% of men achieved the 3-factor control target (*p* < 0.001). Those in the 5th quintile of SES obtained a better 3-factor control target than those in the 1st quintile of SES (women: 10.6% vs. 9.2%, men: 13.4% vs. 11.1%). Women were less likely to achieve the 3-factor control target regardless of the SES index. When adding BMI <30 kg/m^2^ to the 3-factor control target, the patients who achieved it fell to 6.6%, and there were differences between the sexes (Tables 2, 3).

Odds ratios of factors associated with suboptimal control targets in the multilevel analysis are shown in Table 4. The intraclass correlation indicated that the clustering of practices in relation to glycemic control was marginal (intraclass correlation = 0.010).

**TABLE 4:** Factors associated with suboptimal glycemic and multifactorial control targets (aORs and 95% CI).

	Model 1: Suboptimal Glycemic control target OR (95% CI)	Model 2: Suboptimal 3-factor control targets (HbA1c, BP, LDL) OR (95% CI)	Model 3: Suboptimal 4-factor control targets (HbA1c, BP, LDL, & BMI) OR (95% CI)
Sex (male: reference)	0.97 (0.94–1.01)	1.26 (1.19–1.34)	1.46 (1.33–1.59)
**SES index (1st quintile: least deprived, reference)**			
2nd quintile	0.98 (0.90–1.07)	0.79 (0.58–1.08)	0.75 (0.51–1.10)
3rd quintile	1.01 (0.92–1.11)	0.83 (0.59–1.16)	0.83 (0.54–1.27)
4th quintile	1.16 (1.06–1.26)	1.47 (1.04–2.07)	1.41 (0.92–2.15)
5th quintile	1.20 (1.10–1.32)	1.09 (0.77–1.54)	1.11 (0.72–1.70)
Age	0.96 (0.96–0.97)	0.96 (0.96–0.97)	0.96 (0.96–0.97)
Duration of T2DM	1.08 (1.08–1.09)	1.01 (1.00–1.02)	1.00 (0.99–1.00)
Coronary heart disease	1.10 (1.04–1.16)	0.56 (0.52–0.62)	0.66 (0.59–0.75)
Peripheral arteriopathy	1.22 (1.13–1.32)	1.01 (0.88–1.16)	1.11 (0.92–1.35)
Diabetic retinopathy	1.68 (1.53–1.84)	1.44 (1.18–1.75)	1.18 (0.91–1.52)

CI, confidence interval; SES, socioeconomic status.

Faults in achieving glycemic control were associated with being in the 4th and 5th quintiles of SES, and having coronary heart disease, peripheral arteriopathy, and retinopathy was related to not achieving glycemic control. Once we studied the 3-factor and 4-factor control targets (models 2 and 3), we found that women were less likely to reach the 3-factor and 4-factor control targets along with having diabetic retinopathy.

## Discussion

The glycemic control was achieved by 63% of primary care diabetic patients aged 40–75 years in the HHH study. However, only 10.3% achieved the 3-factor control target (HbA1c, BP, and LDL), and 6.6% achieved the BMI <30 kg/m^2^ (4-factor control). Women had better glycemic control (HbA1c) but worst 3-factor and 4-factor control targets regardless of the SES.

In our population, we found that 60.5% of our patients had had their HbA1c checked in the last year. Our results are lower than in Canada, where 68.9% of patients had a baseline HbA1c assessment at a 1-year follow-up (23), or in the United Kingdom, where 69% had all the annual measures during the 5 years (19). In our study, high-SES participants were less likely to have an HbA1c measure, which could be explained by patients in that quintile of SES receiving care outside of the public health system. This contrasts with the UK study where patients belonging to the SES 5th quintile group were more likely not to have annual HbA1c monitoring. Perhaps, due to challenges concerning access to healthcare, this needs additional investigation. In this study, we compared those patients who had at least one measure of HbA1c in the 1-year follow-up and those who did not. Although we have a large sample size, many patients were excluded as they did not meet the inclusion criteria. This study collected information from the clinical practice as information was recorded during the clinical encounter. This approach to using real-world data has limitations as not all the variables were recorded, in contrast to a randomized clinical trial where missing data can be assessed more tightly (24). Other studies with the same aim and size population decided not to impute data (13, 14, 17), although they considered the missing data as a limitation. We focused on analyzing data from the clinical practice to capture how care was delivered, but the quality of the real-world data still has to improve to generate real-world evidence (25).

The glycemic target was achieved in 63.2% of our patients, which is consistent with other studies where only one-half or one-third of cohorts achieved HbA1c <7% (15, 17). Optimal glycemic target has been described in 62.8% of patients in Norway (26), 52.9% in Canada (13), and 46.7% of patients in the United Kingdom (19). Healthcare access and the healthcare provision could explain these differences. By taking into account a global perspective of the disease, health outcomes such as good control of disease may differ by socioeconomic differences. We found that sex and SES were related to the achievement of T2DM targets. The effect of sex on glycemic control has been discussed before without clear findings. Some studies suggested that women were more likely to have suboptimal control (17, 18), but other studies found the opposite (14, 15). Even so, these differences were less than 2% between men and women. In this study, more women reached glycemic targets compared to men (64.2 vs. 62.4%), but sex was not related to optimal glycemic control while the SES index was. The optimal control decreased in both sexes from the 1st quintile to the 5th quintile of SES. Our results are in accordance with Collier et al. (18) and Whyte et al. (19), who showed that greater social deprivation was less likely to reach the glycemic target. These findings caption the importance of addressing social inequalities in people with T2DM to try to improve the glycemic target in those patients most disadvantaged.

Our study found that 10.3% of the patients attained the 3-factor control target with results similar to Wan et al. who registered 9.45%. However, Braga et al. registered that 19% of their patients met the three goals (13), which may be related to differences in participant enrollment. In Spain, Ibáñez et al. published a study in which patients with a lower SES index less frequently reached HbA1c and BP targets, which corresponds with our results (17). In our case, patients who achieved the 3-factor control target more frequently were those in the lowest quintile of SES. This unexpected finding could be explained by patients in the lowest quintile of SES requiring more frequent contact with their primary care centers compared to those in the 1st and 2nd quintiles of SES. Obesity is linked to T2DM (27) and the most deprived quintiles (18). When this risk factor was added, the result was quite poor, only 6.6% of the population achieved the 4-factor control targets.

These findings highlight the need to address whether healthcare outcomes should continue focusing on T2DM management on the HbA1c target as the primary goal or approach it as a 3-factor control target, not only because a few more than half of the patients live with hypertension and obesity but also because guidelines are recommending a 4-factor control target. Improving glycemic control remains a key target to reducing diabetes complications; however, glycemic and especially 3-factor control can be challenging. Moreover, achieving the four targets for a majority of the population is an unrealistic aim. We need more research highlighting the approach to reducing T2DM complications and overall cardiovascular risk factors to understand which targets or combinations of targets are more beneficial for the patients. In contrast, social determinant perspectives, such as the SES index, must be taken into account in developing effective strategies for the management of T2DM. If we address T2DM as an illness where cumulative disadvantage is present (28, 29), we will focus on those groups of patients who are more vulnerable and ensure they receive proper care. Doctors should be trained to address social determinants as they are trained to treat T2DM, but also public policies should take them into account to reduce social inequalities.

### Strengths and limitations

There are several strengths of this study: first, the data source, the electronic health record, is “real-world data” of glucose and other factors management. Second, we addressed diabetes control by focusing on the multifactorial control targets, including BMI. We also highlighted that sex and SES may have a role in the optimal control of glycemic and multifactorial targets in T2DM.

The study was limited by the lack of clinical data from secondary care or private services used by some patients. Lifestyle, treatment, or a number of primary care visits were not collected; these variables could have helped us to interpret our results more clearly. Finally, we have some missing data from some of the variables that were excluded from the analysis. We cannot exclude the possibility of bias because of the missing data that was collected from a retrospective database.

## Conclusion

This study showed that differences in socioeconomic status are related to poorer glycemic control in patients with T2DM. Optimal 3-factor control targets (HbA1c ≤ 7%, LDL < 100 mg/dl, and BP < 140/90 mmHg) were seldom achieved by the diabetic population, and being women was associated with suboptimal 3-factor control of cardiovascular disease risk factors.

## Data availability statement

All data generated or analyzed during this study are included in this published article/Supplementary material. All methods were carried out in accordance with relevant guidelines and regulations. Further inquiries can be directed to the corresponding author.

## Ethics statement

The studies involving human participants were reviewed and approved by the Heart Healthy Hoods study was approved by Madrid Primary Care Research Committee and the Ethics in Research Committee of the Madrid Health Care System (ERC-2013-StG-336893). This work was additionally approved by the Ethics in Research Committee of the Ramón y Cajal Hospital at Madrid city (341/2018). Both Ethics Committees have waived the requirement of the informed consent for this population-based study. Written informed consent for participation was not required for this study in accordance with the national legislation and the institutional requirements.

## Author contributions

SAB, EPC, and IDC: conceptualization, funding acquisition, methodology, supervision, and writing – original draft. SAB and FRC: formal analysis. SAB: investigation. IDC and MF: project administration. SAB, EPC, IDC, FRC, PG, and MF: writing – review and editing. All authors contributed to the article and approved the submitted version.
