# Supplementary material for: Inequalities in glycemic and multifactorial cardiovascular control of type 2 diabetes: The Heart Healthy Hoods study
Source: Front Med (Lausanne). 2022 Dec 7;9:966368. doi: 10.3389/fmed.2022.966368 (PMC9769119; doi:10.3389/fmed.2022.966368)
Supplement: Supplementary file 1 [file Data_Sheet_1.docx]

**Supplementary file 1.** Figure: Participant case selection flow chart


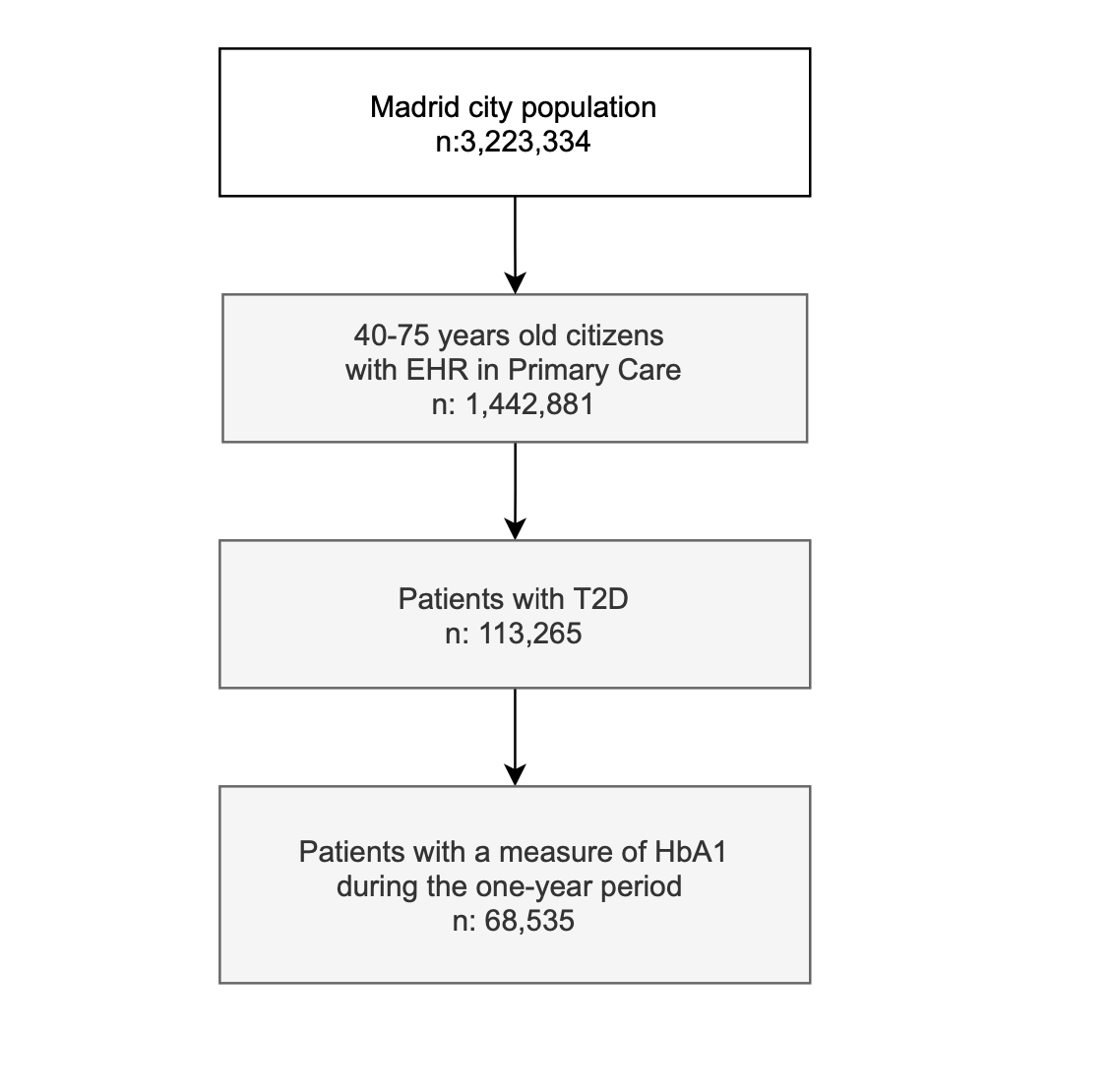


**Supplementary file 2:** Baseline characteristics of the health centres.

| Primary Health Centre Characteristics: |  |  |
| --- | --- | --- |
| Number of Family doctors | Mean, standard deviation | 17,06 (6,60) |
| Number of Nurses | Mean, standard deviation | 15,57 (5,96) |
| Daily consultations of family doctors (patients/ day) | Mean, standard deviation | 32.84 (2.45) |
| Daily consultations of nurses  (patients/day) | Mean, standard deviation | 20.59 (1.77) |

**Supplementary file 3:** Patients characteristics with and without HbA1c.

|  |  | Patients without HbA1  n:44730 | Patients with  HbA1  n:68535 | p-value |
| --- | --- | --- | --- | --- |
| Age | Mean, sd | 61.5(9.2) | 62.6(8.7) | <0.001 |
| Duration of DM2 (years) | Mean, sd | 9.33(5.7) | 9.43(5.9) | 0.006 |
| Sex (male) | n (%) | 27300(61.0) | 38955(56.8) | <0.001 |
| Foreigners |  | 3578 (8.0) | 4111(6.0) | <0.001 |
| Socioeconomic Status index: |  |  |  | <0.001 |
| 1^st^ quintile | n (%) | 11239 (25.3) | 14764 (21.6) |  |
| 2^nd^ quintile | n (%) | 8415 (18.9) | 12405 (18.2) |  |
| 3^rd^ quintile | n (%) | 8470 (19.0) | 13110 (19.2) |  |
| 4^th^ quintile | n (%) | 9004 (20.2) | 15393 (22.6) |  |
| 5^th^ quintile | n (%) | 7364 (16.6) | 12568 (18.4) |  |
| Cardiovascular Risk Factors: |  |  |  |  |
| Tobacco | n (%) | 1490 (18.6) | 4799 (18.0) | 0.20 |
| Dyslipidaemia | n (%) | 24474 (54.7) | 43135 (62.9) | <0.001 |
| Hypertension | n (%) | 26868 (60.1) | 43384 (63.3) | <0.001 |
| Obesity | n (%) | 6197(48.0) | 18407(46.1) | <0.001 |
| Clinical Parameters: |  |  |  |  |
| SBP (mmHg) | Mean, sd | 138.4 (18.0) | 139.5 (16.9) | <0.001 |
| DBP (mmHg) | Mean, sd | 79.2 (10.4) | 80.4 (10.0) | <0.001 |
| BMI (kg/m2) | Mean, sd | 30.4 (5.6) | 30.2 (5.2) | <0.001 |
| LDL (mg/dL) | Mean, sd | 104.0 (35.3) | 103.2 (32.7) | 0.22 |
| HDL (mg/dL) | Mean, sd | 48.0 (13.5) | 48.9 (12.9) | <0.001 |
| Estimated Glomerular Filtration, (mL/min/1,73 m^2^) | Median, IQR | 83.8 (63.2, 90.9) | 86.8 (70.0, 91.7) | <0.001 |
| Albuminuria (mg/g) | Mean, sd | 83.8 (63.2, 90.9) | 48.9 (310.3) | 0.005 |
| T2DM Complications: |  |  |  |  |
| Coronary heart disease | n (%) | 5389 (12.0) | 7057 (10.3) | <0.001 |
| Stroke | n (%) | 2801 (6.3) | 3908 (5.7) | <0.001 |
| Peripheral Arteriopathy | n (%) | 2335 (5.2) | 3212 (4.7) | <0.001 |
| Renal Chronic Disease* | n (%) | 69 (14.1) | 1910(11.7) | 0.10 |
| Diabetic Nephropathy | n (%) | 105 (21.0) | 2232(14.5) | <0.001 |
| Retinopathy | n (%) | 1507 (3.4) | 2127 (3.1) | 0.013 |

Sd: Standard deviation. SBP: Systolic blood pressure. DBP: Diastolic blood pressure, BMI: body mass index

*The percentages and number of patients were made with patients who had at least two values of estimated glomerular filtration less than 60 mL/min/1,73 m^2^

**Supplementary file 4:** Characteristics of cardiovascular factors of men and women.

*: Total and %

| FEMALE | All | 1^st^ Quintile  n:5903 | 2^nd^ Quintile  n:5231 | 3^rd^ Quintile  n:5777 | 4^th^ Quintile  n:6768 | 5^th^ Quintile  n:5768 | p-value |
| --- | --- | --- | --- | --- | --- | --- | --- |
| Tobacco* | 1485 (13.0) | 327 (13.8) | 294 (14.1) | 261 (11.5) | 317 (12.0) | 281 (13.6) | 0.027 |
| Dyslipidaemia* | 19446 (65.7) | 3968 (67.2) | 3358 (64.2) | 3930 (68.0) | 4359 (64.4) | 3761 (65.2) | <0.001 |
| Hypertension* | 19873 (67.2) | 3776 (64.0) | 3428 (65.5) | 3975 (68.8) | 4619 (68.2) | 3980 (69.0) | <0.001 |
| Obesity* | 9002 (48.9) | 1532 (47.1) | 1494(47.9) | 1670(46.9) | 2290 (53.1) | 1986(53.4) | <0.001 |
| MALE |  |  |  |  |  |  |  |
|  | All | **1^st^ Quintile**  n:8861 | **2^nd^ Quintile**  n:7174 | **3^rd^ Quintile**  n:7333 | **4^th^ Quintile**  n:8625 | **5^th^ Quintile**  n:6800 |  |
| Tobacco* | 3314 (21.8) | 700 (19.7) | 632 (21.2) | 594 (20.7) | 717 (21.4) | 664 (27.5) | <0.001 |
| Dyslipidaemia* | 23689 (60.8) | 5511 (62.2) | 4116 (57.4) | 4594 (62.6) | 5137 (59.6) | 4233 (62.3) | <0.001 |
| Hypertension* | 23511 (60.4) | 9570 (60.3) | 7306 (59.1) | 7560 (60.9) | 8145 (58.0) | 6464 (57.4) | <0.001 |
| Obesity* | 9405 (51.09) | 1926 (41.7) | 1706(41.8) | 1675(40.6) | 2276 (45.0) | 1789(44.9) | <0.001 |
